# Reform of emergency services: immediate effects on cardiac care unit and ICU patient intake

**DOI:** 10.1186/cc12213

**Published:** 2013-03-19

**Authors:** AO Alaspää, T Valpas, V Rautava, A Palomäki

**Affiliations:** 1 Kanta-Häme Central Hospital, Hämeenlinna, Finland

## Introduction

Early onset effective care in the emergency department (ED) has been reported to have a great influence on the intensive care patients' morbidity and mortality [1]. Little is known about the influence of the reorganisation of the ED on patient intake to the ICUs. The aim of this study was to analyse monthly intake of patients from the ED to the cardiac care unit (CCU) and ICU before and after the reform of emergency services.

## Methods

In Kanta-Häme Central Hospital, a new ED started on 1 April 2007. Four older emergency rooms were combined into one bigger emergency department and an observation ward was introduced with continuous follow-up of vital signs. This study is a retrospective analysis of the patient intake to the CCU and ICU 1 year before and after the reorganisation. Using as data the Finnish Intensive Care Quality Consortium (Intensium, Finland) database and the cardiac database of the hospital, patient transfer from ED to the ICU and CCU was collected and analysed. Monthly pre/post comparisons were carried out statistically by a nonparametric Wilcoxon signed-rank test.

## Results

**Figure 1:**
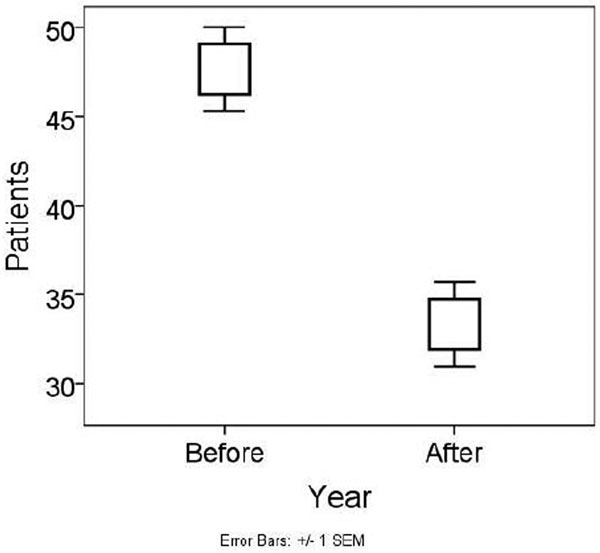
**Combined patient flow to the ICU and CCU before and after the reformation of the ED**.

The total decrease in monthly patient inflow from ED to the ICU and CCU was 30.1% (*P *= 0.003); that is, from the mean of 47.7 ± 8.2 to 33.3 ± 8.3 patients (Figure 1). The 7.1% decrease in patients taken into the ICU (12.9 ± 4.1 to 12.0 ± 4.6 patients) was not statistically Significant. However, the intake from the ED to the ICU decreased by 38.6% (from 34.8 ± 6.5 to 21.3 ± 5.1 patients) (*P *= 0.002).

## Conclusion

According to our results the reform of the ED may have a significant role in the total inflow of patients to the CCU and ICU.
